# Hepatocellular carcinoma after prior sorafenib treatment: incidence, healthcare utilisation and costs from German statutory health insurance claims data

**DOI:** 10.1186/s13561-018-0199-1

**Published:** 2018-08-27

**Authors:** Johannes Clouth, Astra M. Liepa, Guido Moeser, Heiko Friedel, Magdalena Bernzen, Jörg Trojan, Elena Garal-Pantaler

**Affiliations:** 1 Medical Affairs, Lilly Deutschland GmbH, Werner-Reimers-Str. 2-4, 61352 Bad Homburg, Germany; 2 Eli Lilly and Company, Lilly Corporate Center, Indianapolis, IN 46285 USA; 3 masem Research Institute GmbH, Unter den Eichen 5/G, D-65195 Wiesbaden, Germany; 4 Team Gesundheit GmbH, Rellinghauser Straße 93, 45128 Essen, Germany; 5 Universitätsklinikum Frankfurt, Medizinische Klinik 1, Theodor-Stern-Kai 7, 60590 Frankfurt, Germany

**Keywords:** Hepatocellular carcinoma, Germany, Second line, Sorafenib, Health economics, German statutory health insurance claims data

## Abstract

**Objective:**

To estimate both the number of patients with hepatocellular carcinoma (HCC) eligible annually for second-line therapy following sorafenib in Germany and the healthcare costs accrued by patients meeting eligibility criteria.

**Methods:**

Patients with an HCC diagnosis and one or more sorafenib prescription were identified from samples of > 3 million insured persons in each of 2012, 2013 and 2014 using the anonymised Betriebskrankenkasse health insurance scheme database. Incidence rates from 2013 were extrapolated to the German population using data from the statutory health insurance system database and Robert Koch Institute. Resource use and cost data were collected for a subset of patients with follow-up data post-sorafenib.

**Results:**

Between 1032 and 1484 patients with HCC in Germany (893–1390 publicly insured patients) were estimated as likely to be eligible for second-line therapy after sorafenib annually. For post-sorafenib analyses, 117 patients were identified with HCC, one or more sorafenib prescription and considered potentially eligible for second-line treatment, 15 of whom were alive after 12 months’ follow-up. Total mean costs per patient accrued in the 12 months after sorafenib treatment ended were €11,152 (hospital care, €6483 [58.1%]; outpatient prescriptions, €3137 [28.1%]).

**Conclusion:**

The estimated number of publicly insured HCC patients annually eligible for second-line therapy in Germany was < 1400 and mean total costs accrued in the year after completion of sorafenib therapy were approximately €11,000 per patient for the German statutory healthcare system. These estimates can be used when evaluating the budgetary impact of new second-line therapies for advanced HCC in Germany.

**Electronic supplementary material:**

The online version of this article (10.1186/s13561-018-0199-1) contains supplementary material, which is available to authorized users.

## Background

Liver cancer is one of the most common cancers worldwide (annual incidence: 10.1 cases per 100,000), with 70.8% of cases occurring in men [1]. The prognosis of liver cancer is poor, and it is the second most common cause of cancer-related deaths worldwide [1]. Hepatocellular carcinoma (HCC) accounts for approximately 70–85% of the total liver cancer burden worldwide [2]. Around 80% of HCC cases are associated with chronic hepatitis B or C viral infections [3, 4]. However, the risk of HCC is increased in diabetic populations, particularly those with type 2 diabetes mellitus [5], as well as in patients with the metabolic syndrome who can present with non-alcoholic steatohepatitis and no cirrhosis [6, 7]. Since the metabolic syndrome is highly prevalent, even small increases in obesity- or diabetes-related risk could result in a large number of HCC cases [8]. In Germany, the age-adjusted incidence rates of liver cancer in 2012 [1] and 2013 [9], respectively, were 2.3 and 3.5 per 100,000 population in females and 7.2 and 10.3 per 100,000 population in males; crude rates were 6.4 per 100,000 population in females and 15.6 per 100,000 population in males in 2013 [9]. Recent estimates of HCC incidence in Germany were not identified in the literature.

Staging of HCC is important for determining prognosis and planning therapy and includes an assessment of tumour stage, underlying liver function and clinical performance status. A number of different staging systems are used in HCC, although the Barcelona Clinic Liver Cancer (BCLC) classification is the only system that takes into account all of these factors and is endorsed for prognostic prediction and treatment allocation [10, 11]. Treatment options for HCC include surgical resection, liver transplantation and local ablation for early-stage disease, locoregional treatments (e.g. transarterial chemoembolisation and radioembolisation) for intermediate-stage disease, and systemic therapies for advanced-stage disease. Current treatment guidelines recommend that patients with advanced HCC (BCLC stage C; defined as disease with vascular invasion or metastases) should receive sorafenib [10, 12]. Best supportive care or the inclusion of patients in clinical trials after disease progression or intolerance to sorafenib is recommended [10, 12], as there are no approved second-line treatments for HCC at the time of these analyses.

In Germany, incidence and prevalence data are required to assess the potential benefits and financial impact of any new treatment according to the Act on the Reform of the Market for Medicinal Products [13]. Currently, the size of the potential population of patients with advanced HCC eligible for second-line therapy is unknown as relevant epidemiologic data are not available. Thus, the present study was performed to estimate the number of patients with advanced HCC eligible for second-line therapy annually in Germany and the healthcare costs accrued by patients with HCC eligible for second-line therapy based on data from the German statutory health system.

## Methods

### Study design and data source

This was a retrospective, observational study of data from the Betriebskrankenkasse (BKK) health insurance scheme in Germany over a 7-year period (2008 to 2014). The BKK includes data from insured persons registered at company health insurance funds. Although initially for employees of companies or enterprises, these funds have been open to all German citizens since 1996 and have become more representative of the entire statutory health insurance scheme. We requested and obtained access to these strictly regulated data from the BKK who had no other involvement in these analyses. After BKK approval, data from the electronic databases of six different anonymised statutory health insurance funds were made available from central data service providers (Bitmarck Service for BitInfonet, and Bitmarck-Beratung for ISKV 21c) and participating company health insurance funds directly. These data were gathered under naturalistic conditions and anonymised by the providers in accordance with an approved data privacy concept. The raw data were then imported, prepared and checked by the authors using previously established processes.

The target patient population, i.e. patients with advanced HCC who had received sorafenib and were potentially eligible for second-line therapy, was identified. The data extracted for the present study were demographic information, utilisation of health services (e.g. hospitalisation, inpatient and outpatient care, prescriptions, sickness and other benefits) and associated costs. No ethics approval or consent to use the included information was required because of the anonymised nature of all data.

### Identification of the target populations

The source sample population consisted of > 3 million insured persons in each of the study index years (2012 to 2014) and was representative of the German statutory health insurance population in terms of gender and age group distributions (Additional file 1: Table S1).

**Fig. 1:**
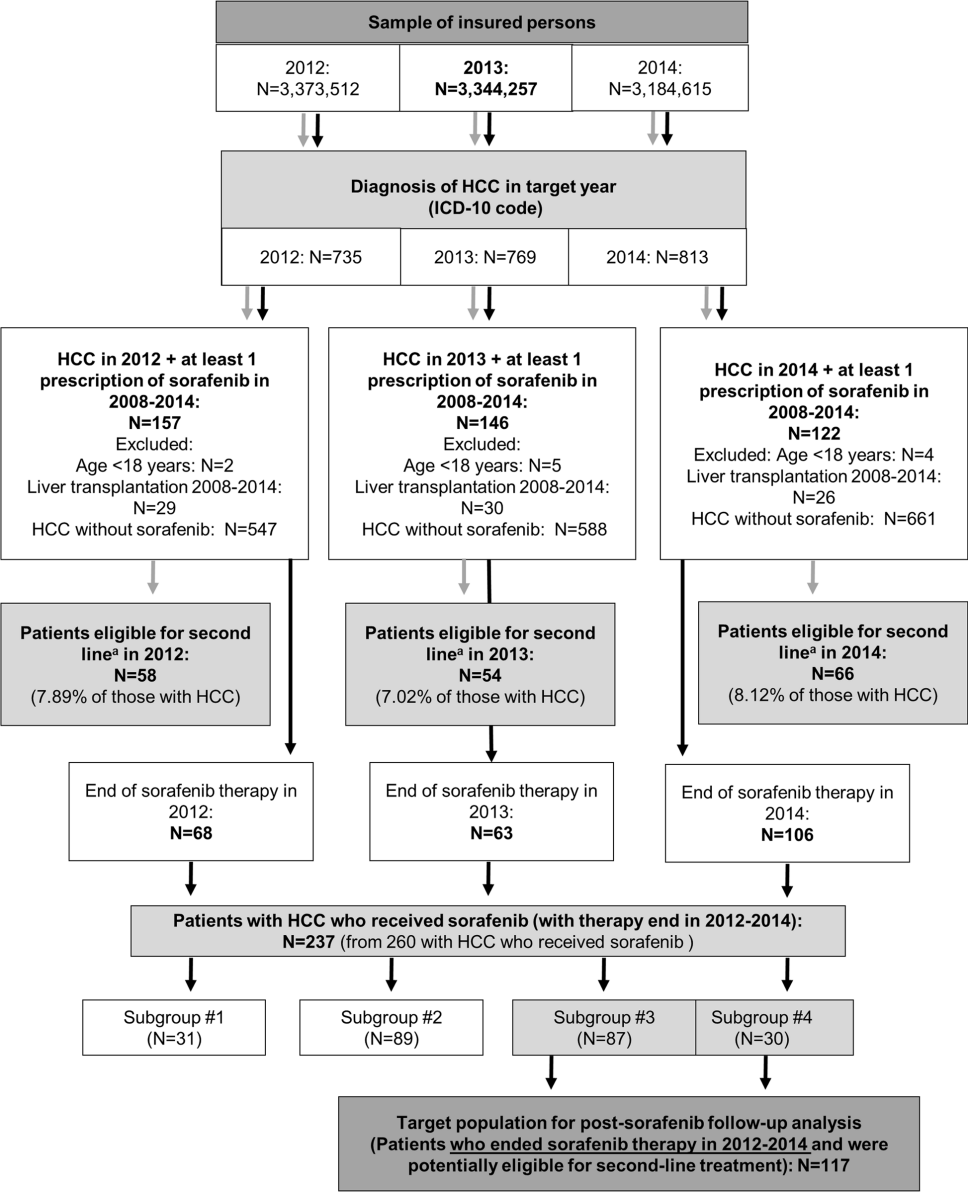
Flowchart of HCC patient selection for epidemiological analysis and post-sorafenib follow-up analysis. Subgroup #1: patients still alive at the end of the observation period but with fewer than 70 observation days from the last sorafenib prescription; Subgroup #2: patients who died while on sorafenib therapy; Subgroup #3: patients who died within the observation period but not under sorafenib therapy (i.e. more than 70 days between the last sorafenib prescription and death); Subgroup #4: patients who were still alive at the end of the observation period and for whom at least 70 days had passed between the last prescription of sorafenib and the end of the observation period. ^a^Patients alive after sorafenib therapy end/failure. *HCC* hepatocellular carcinoma, *ICD-10* International Classification of Diseases and Related Health Problems, tenth revision

Two different approaches were used to identify target populations so that the two study objectives could be met. The first approach identified all patients with advanced HCC potentially eligible for second-line therapy during the analysis period. The second approach identified patients with the required follow-up data to allow estimation of healthcare costs accrued by patients with HCC eligible for second-line therapy. The details are shown in the study flowchart (Fig. 1), with the two methodological approaches indicated with orange and blue arrows.

First, to estimate the number of patients with advanced HCC eligible for second-line therapy after sorafenib (Fig. 1, orange arrows), patients with a diagnosis of liver cancer, and HCC in particular, were identified using the *International Classification of Diseases and Related Health Problems, tenth revision* (ICD-10) codes C22 and C22.0, respectively, every year from 2012 to 2014 (each was considered an index year). Diagnoses made in the inpatient and/or ambulatory care setting were permitted. Inpatients with HCC were identified using primary or secondary inpatient C22.0 diagnoses, whereas outpatients with HCC were identified using the ‘assured’ (gesichert) and ‘status after’ (“Zustand nach”) C22.0 diagnosis. Patients who had no relevant ICD-10 diagnosis during 1 year preceding the index year were considered incident in the corresponding index year. Patients younger than 18 years (in the corresponding index year) and those who had undergone liver transplantation at any time over the study period were excluded. Patients with a diagnosis of HCC together with at least one prescription for sorafenib in each index year were then identified. Sorafenib prescriptions were identified using ATC code L01XE05 for outpatient prescriptions and OPS codes 6–003.b* for inpatient treatment. Patients who were alive after the end of sorafenib therapy for each index year were considered to be the target population, i.e. patients with advanced HCC previously treated with sorafenib who were eligible for second-line therapy.

To obtain a population with sufficient follow-up data post-sorafenib therapy for determination of healthcare costs (Fig. 1, blue arrows), we identified patients with a diagnosis of HCC who had received at least one prescription for sorafenib and who had ended sorafenib therapy in the timeframe 2012 to 2014. No maximum end date for sorafenib therapy in 2014 was designated, acknowledging that some patients may have less follow-up data. Clinically accepted markers for identifying treatment failure either could not be identified from the database because of missing or unspecific coding (i.e. radiologic findings or symptomatic progression) or were thought to be unreliable (i.e. discontinuation of therapy following treatment failure). We therefore performed a series of exploratory analyses to identify the target group of patients who had failed sorafenib therapy and who were possible candidates for second-line therapy (Additional file 2). An explorative approach was used that combined potentially distinguishing markers to detect failure of first-line therapy. Markers considered were the frequency and duration of sorafenib prescriptions, the pattern of sorafenib prescriptions (i.e. full dose, dose reductions and dose interruptions), observation time and adverse events. The data were stratified to examine potential influences of, and differences between, these markers and to find an adequate definition of treatment failure. From these analyses, the following four subgroups were identified:

- Subgroup #1: Patients still alive at the end of the observation period (31 December 2014) and with fewer than 70 observation days (allowing for a 56-day intake of a reduced dosage [two tablets daily instead of four tablets daily as is recommended [14]] plus 14 days of follow-up) from the last sorafenib prescription.
- Subgroup #2: Patients who died while on sorafenib therapy.
- Subgroup #3: Patients who died within the observation period (i.e. before 31 December 2014) and no longer received sorafenib therapy (i.e. more than 70 days of follow-up between the last sorafenib prescription and death).
- Subgroup #4: Patients who were still alive at the end of the observation period (31 December 2014) and for whom at least 70 days of follow-up had passed between the last prescription of sorafenib and the end of the observation period.

Patients in subgroup #1 had insufficient follow-up to allow meaningful analysis. Patients in subgroup #2 died while on sorafenib treatment and could not have received a second-line agent. Therefore, subgroups #1 and #2 were not considered for further analysis. Patients in subgroups #3 and #4 were considered to have failed first-line sorafenib therapy and were potentially eligible for second-line treatment and therefore constituted the target population for post-sorafenib follow-up analysis.

### Data collection

Data from the target population for post-sorafenib follow-up analysis were examined retrospectively for up to 4 years from the first prescription of sorafenib to identify pre-existing and concomitant conditions diagnosed in the inpatient and/or outpatient settings, and especially risk factors for HCC (e.g. cirrhosis of the liver, hepatitis B and C). Treatments received prior to initiation of sorafenib therapy in inpatient and/or outpatient care were also captured.

Starting from the end of sorafenib therapy (defined as last prescription + 56 days) and until 31 December 2014 or death, resource use (i.e. hospital stays and care, including ambulatory treatments; outpatient visits; outpatient prescriptions; work disability; remedies; and other benefits) and associated cost data were gathered for the target population. Cost data were for all healthcare utilisations classified into the listed resource use categories that had been refunded or paid by the sickness funds. A so called “Orientierungspunktwert” (reference point value) according to the Uniform Evaluation Scale catalogue, also known as EBM (Einheitlicher Bewertungsmaßstab), was used for monetarisation of patient-physician contacts in outpatient care. Survival data from the last sorafenib prescription were also collected.

### Data analysis

Crude incidence and prevalence rates of HCC for 2013 in Germany were estimated using data from two different sources:

(i) German statutory health insurance system database (Gesetzliche Krankenversicherung [GKV]) (2013) and
(ii) Robert Koch Institute (2013) [9, 15].

Calculations were based on the annual number of HCC diagnoses reported for the stated year. The crude incidence rate of HCC was calculated as the number of new disease diagnoses/sample population × 100,000 [16].

To calculate the crude incidence rate for the GKV, the study sample population (BKK) estimates underwent age- and gender-adjusted extrapolation to the total GKV population using published methods [17].

The Robert Koch Institute reported only diagnoses of liver cancer (C22). Therefore, the number of HCC cases reported by the Robert Koch Institute was estimated using two sources: the first based on the Robert Koch Institute report that 66% of liver cancers in Germany are HCC [15] and the second based on a published paper that reported a value of 80% [18]. Numbers for the total population in Germany, used for estimating crude incidence rates, were as stated by the German Federal Statistical Office/eurostat [19, 20].

The mean (standard deviation; 95% confidence interval [CI]) proportion of patients in the BKK with HCC eligible for second-line treatment from each of the index years (2012 to 2014) was calculated and then applied to the estimated populations of patients with HCC in Germany as identified from the two sources mentioned above.

Resources and costs were analysed from the perspective of the German statutory healthcare system (i.e. public healthcare provider). Mean real (unweighted) estimates of resource use and costs were calculated for the target population for each type of resource for the first year after the end of sorafenib therapy. Data from all eligible insured patients were included, independent of individual length of follow-up i.e., the yearly costs of the target population were evaluated from the payer perspective. Utilisation of inpatient care was measured from the documented admission date and included ambulatory and hospital treatments. All costs were expressed in euros, using the values recorded without correction.

## Results

### Incidence and prevalence

The numbers of patients with diagnoses of HCC in our study population in 2012 to 2014 are shown in Fig. 1 (orange arrows); 769 such diagnoses were recorded in 2013. The estimated crude incidence and prevalence rates of HCC for the present sample population in 2013 and the total GVK in 2013, as well as crude estimates for the German population based on data from the Robert Koch Institute, are shown in Table 1 [9].

**Table 1:** Estimated incidence and prevalence data for HCC in Germany in 2013

Population/parameter	Total
Study sample population (insured for ≥1 day in 2013)	3,344,257
New HCC diagnoses	407
Crude HCC incidence (per 100,000)	12.17
HCC diagnoses (throughout 2013)	769
Crude HCC prevalence (per 100,000)	22.99
Proportion of population with HCC eligible for second-line therapy, mean % (95% CI)^a^	7.68% (7.02–8.33)
German statutory health system population (at 1 July 2013)	69,854,922
New HCC diagnoses (extrapolated incidence)^b^	8841
Crude HCC incidence (extrapolated) (per 100,000)^b^	12.66
HCC diagnoses (extrapolated throughout 2013)	16,685
Crude HCC prevalence (extrapolated) (per 100,000)^b^	23.89
Number eligible for second-line therapy, low–high limit^c^	1171–1390
Germany – RKI (2013)	
New HCC diagnoses^d^	5801–7032
Crude HCC incidence (per 100,000)^e^	7.18–8.71
HCC diagnoses (estimated throughout 2013)^f^	14,697–17,814
Crude HCC prevalence (estimated throughout 2013) (per 100,000)^f^	18.20–22.06
Number eligible for second-line therapy, low–high limit^g^	1032–1484

*C22* International Classification of Diseases, tenth revision code for liver cancer, *CI* confidence interval, *HCC* hepatocellular carcinoma, *RKI* Robert Koch Institute

^a^Mean of estimates for 2012 (7.89% [58/735]), 2013 (7.02% [54/769]) and 2014 (8.12% [66/813])

^b^Patients with a diagnosis of HCC in 2013, extrapolated from the German Betriebskrankenkasse (BKK) health insurance scheme data (present study sample), age- and gender-adjusted to the entire German statutory health insurance system

^c^Based on 95% CI for study sample estimate (i.e. 7.02–8.33)

^d^Assumes that HCC accounts for 66–80% of total liver cancer burden (RKI estimate [15] – reported value in literature [18]) and based on total number of new liver cancer cases reported in 2013 by the RKI (male: 6160; female: 2630)

^e^Calculated using number of new HCC cases estimated to be reported in 2013 by the RKI [9] as a proportion of the German population reported by the German Federal statistical office/eurostat [19, 20] in 2013 (*N* = 80,767,463)

^f^Prevalence of insured patients with C22 diagnosis was the sum of 5-year prevalence in 2012 (*n* = 10,800) and incidence in 2013 (*n* = 8790) = 19,590. Since the 10-year prevalence of C22 reported in 2013 (*n* = 14,990) exceeded the 5-year prevalence for the same year (*n* = 12,010) by 1.248 times, this factor (1.248) was used to estimate 10-year prevalence in 2012 and correct calculations as follows: NC22 in Germany = (10,800 × 1.248 = 13,478) + 8790 = 22,268 individuals with C22 diagnosis in the course of 2013. Corrections to estimate HCC prevalence were based on the assumption that HCC accounts for 66–80% of total liver cancer burden (RKI estimate – reported value in literature [18])

^g^Assumes that HCC accounts for 66–80% of the total liver cancer burden (RKI estimate – reported value in literature [18]) and 95% CI for study sample estimates (i.e., 7.02–8.33)

The mean (standard deviation; 95% CI) proportion of the study population with HCC that was eligible for second-line therapy, based on findings from 2012 to 2014, was 7.68% (0.58; 7.02–8.33). Extrapolating this to the total German population, we estimated that, annually, between 1171 and 1390 patients with HCC were potentially eligible for second-line therapy based on the statutory health system (GKV) data, and between 1032 and 1484 patients were eligible based on Robert Koch Institute data (Table 1). As 86.5% of the population in Germany is covered by public health insurance [19–21], the Robert Koch Institute value would decrease to between 893 and 1284 for publicly insured patients.

### Post-sorafenib follow-up analysis

#### Target population

For the post-sorafenib follow-up analysis, we identified 237 patients with a diagnosis of HCC who had received at least one prescription for sorafenib and who had ended sorafenib therapy in the timeframe 2012 to 2014 (Fig. 1; blue arrows). Of these 237 patients, 31 were in Subgroup #1 and 89 were in Subgroup #2; these patients were not considered further. The target population for post-sorafenib follow-up analysis was the remaining 117 patients in subgroups #3 (*n* = 87) and #4 (*n* = 30) who were considered to have failed first-line sorafenib therapy and were potentially eligible for second-line treatment. The baseline characteristics, comorbidities (including diagnoses of conditions considered to be risk factors) and pre-existing medications of these 117 patients are shown in Table 2.

**Table 2:** Available patient and disease characteristics for patients with HCC potentially eligible for second-line therapy

	Subgroups #3 and #4	Subgroup #3^a^	Subgroup #4^b^
N	117	87	30
Sex, n (%)			
Men	100 (85)	75 (86)	25 (83)
Women	17 (15)	12 (14)	5 (17)
Age, years			
Mean	71.87	71.52	72.90
Pre-existing confirmed diagnoses (ICD code)^c^, n (%)			
Hypertension (I10.x)	96 (83)	70 (81)	26 (53)
Diabetes mellitus (E11, E14)	66 (56)	50 (58)	16 (53)
Liver cirrhosis (K74.3, K74.4, K74.5, K74.6, K70.3, K71.7, P78.8)	62 (53)	45 (52)	17 (57)
Liver cirrhosis and fibrosis (K74.x)	57 (49)	41 (47)	16 (53)
Other liver diseases (K76.x)	46 (39)	30 (26)	16 (53)
Disorders of lipoprotein metabolism and other lipidaemias (E78.x)	51 (44)	35 (40)	16 (53)
Disorders of refraction and accommodation (H52.x)	46 (39)	31 (36)	15 (50)
Obesity (E66.x)	39 (33)	26 (30)	13 (43)
Dorsalgia (M54.x)	37 (32)	28 (32)	9 (23)
Gastritis and duodenitis (K29.x)	35 (30)	28 (32)	7 (23)
Chronic ischemic heart disease (I25.x)	36 (31)	26 (30)	10 (33)
Disorders of purine and pyrimidine metabolism (E79.x)	32 (27)	24 (28)	8 (27)
Cataract (H25.x)	26 (22)	19 (22)	7 (23)
Pre-existing medications (ATC code)^c^, n (%)			
β-blocking agents (C07A)	69 (59)	54 (62)	15 (50)
Loop diuretics (C03C)	45 (38)	36 (41)	9 (30)
ACE inhibitors, plain (not in combination) (C09A)	45 (38)	31 (36)	14 (47)
Lipid-modifying agents, plain (C10A)	33 (28)	21 (24)	12 (40)
Antithrombotic agents (B01A)	33 (28)	24 (28)	9 (30)
Agents for peptic ulcer and gastro-oesophageal reflux disease (A02B)	64 (55)	46 (53)	18 (60)
Anti-inflammatory and antirheumatic products, non-steroids (M01A)^d^	42 (36)	31 (36)	11 (37)
Other analgesics and antipyretics (N02B)	35 (30)	26 (30)	9 (30)
Opioids (N02A)	17 (15)	15 (17)	2 (7)
Antibacterials for systemic use (J01)^e^	41 (35)	30 (34)	11 (37)
Diagnostic agents (V04B, V04C)	33 (28)	25 (29)	8 (27)
Anti-gout preparations (M04A)	31 (26)	23 (26)	8 (27)
Antidiabetes medications, excl. insulins (A10B)	29 (25)	22 (25)	7 (23)
Patients who received chemotherapy prescriptions^f^ (L01)	2 (2)	1 (1)	1 (3)
Mean duration of sorafenib therapy, days		223.6	230.0
Standard deviation		307.3	376.0
Range		28–1630	28–1972

*ACE* angiotensin-converting enzyme, *ATC* anatomical therapeutic chemical, *ICD* International Classification of Diseases

^a^Patients who died during the observation period but not on sorafenib therapy (i.e. more than 70 days between the last sorafenib prescription and death)

^b^Patients who were still alive at the end of the observation period and at least 70 days had passed between the last prescription of sorafenib and the end of the observation period

^c^During the year prior to sorafenib therapy (diagnoses during the quarter in which patients were identified were not included but those of prior quarters were); reported by at least 20% of patients in at least two of the three groups (study population, subgroup #3, subgroup #4), with the exception of opioids, which were an important contributor to the analgesic category

^d^Value may be underestimated because many of these medications are available without prescription (i.e. over the counter)

^e^Patients may have received more than one cycle of treatment with antibiotics

^f^During the year prior to sorafenib therapy

The mean (standard deviation) duration of follow-up of the target population was 170.4 (202.8) days (median 90 days, range 15–1043); 15 patients were still alive after 12 months of follow-up.

#### Resource utilisation and costs

**Fig. 2:**
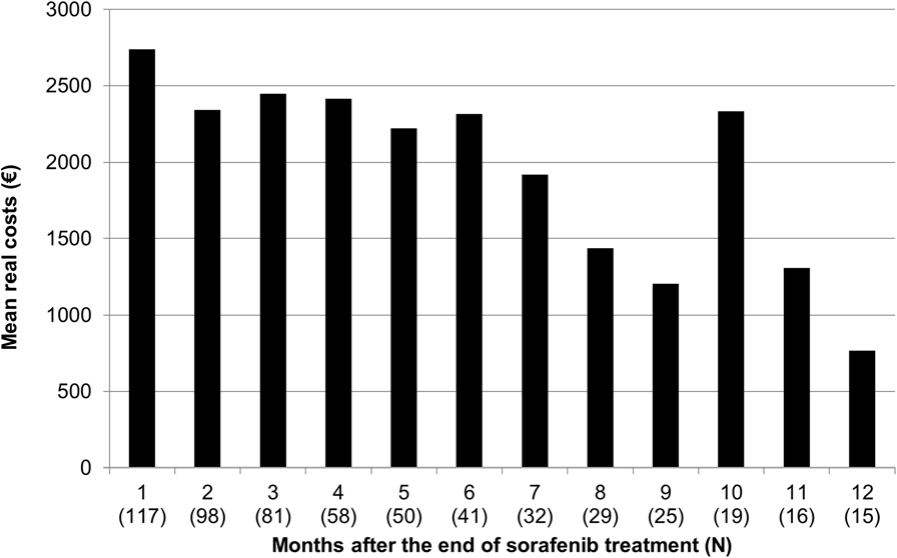
Monthly costs^a^ for patients with HCC previously treated with sorafenib (subgroups #3^b^ and #4^c^). ^a^At values reported for each index year 2012 to 2014; all patients who were insured for at least one day in the corresponding month contributed to costs. ^b^Patients who died within the observation period but not under sorafenib therapy (i.e. more than 70 days between the last sorafenib prescription and death). ^c^Patients who were still alive at the end of the observation period and for whom at least 70 days had passed between the last prescription of sorafenib and the end of the observation period. *HCC* hepatocellular carcinoma

A summary of mean resource use and costs for the 12 months after the end of sorafenib treatment for the target population is presented in Table 3. Mean costs accrued by month in the target population are shown in Fig. 2.

**Table 3:** Resource use and costs for patients with HCC potentially eligible for second-line therapy^a^

Variable	Subgroup #3^b^		Subgroup #4^c^		Subgroup #3 and #4	
	[*n* = 87]		[*n* = 30]		[*n* = 117]	
	Utilisation, n (SD)	Cost per patient (€)	Utilisation, n (SD)	Cost per patient (€)	Utilisation, n (SD)	Cost per patient (€)
Hospital care^d^	1.86 (2.37)	6556.25	1.30 (1.58)	6271.95	1.72 (2.20)	6483.36
Outpatient visits	19.05 (19.43)	862.54	26.77 (23.11)	789.69	21.03 (20.61)	843.86
Outpatient prescriptions	24.94 (24.41)	1451.77	27.90 (27.56)	8025.81	25.70 (25.17)	3137.42
Other benefits^e^	5.60 (6.75)	671.12	5.37 (11.12)	730.96	5.54 (8.04)	686.46
Remedies	0.03 (0.18)	0.64	0.00 (0.00)	0.00	0.03 (0.16)	0.48
Total	–	9542.32	–	15,818.41	–	11,151.58

Data are presented as mean (standard deviation, SD) values

*HCC* hepatocellular carcinoma

^a^Resource utilisation and costs in the 12 months after the end of sorafenib treatment

^b^Patients who died within the observation period but not under sorafenib therapy (i.e. more than 70 days between the last sorafenib prescription and death)

^c^Patients who were still alive at the end of the observation period and at least 70 days passed between the last prescription of sorafenib and the end of the observation period

^d^Costs include all hospital-related costs – admissions and ambulatory treatments in hospital

^e^Additional benefits paid by the German statutory health insurance (e.g. statutory pension fund benefits, therapeutic appliances, travelling costs, nursing care benefits, home healthcare)

In the target population, mean total costs in the 12 months after the end of sorafenib therapy were €11,152 per patient. Hospital care and outpatient prescriptions were the two highest contributing cost groups to the total, accounting for 58.1% (€6483) and 28.1% (€3137) of the total mean costs per patient over the 12-month period, respectively. Mean hospital care costs were also among the highest contributing cost groups for subgroups #3 and #4 when analysed separately, accounting for 68.7% of total mean costs per patient (€6556; *n* = 87) in subgroup #3 and 39.6% of the total mean costs per patient (total costs €6272; *n* = 30) in subgroup #4 over the 12-month period. Mean costs of prescriptions accounted for 50.7% (€8026) of the total costs per patient in subgroup #4 but only for 15.2% (€1452) in subgroup #3 over the 12-month period.

Monthly mean total costs for both subgroups generally declined over time during the first 12 months after the end of sorafenib treatment, with some spikes in cost observed at arbitrary months (Fig. 2). When examining subgroup #4 in more detail (*n* = 30), as this subgroup had longer follow-up data available, there was a general downward trend over time following sorafenib treatment that was reflected in all areas of the statutory health system. Mean monthly costs of hospital care per patient were €2044.89 in the first month and fluctuated between €8 and €3051 in the first 12 months; the costs for appointments with physicians in the outpatient setting were €121 in the first month and declined to €66 in month 12. Similarly, the mean number of monthly prescriptions per patient was 4.20, costing €1412 in the first month, and 4.50 prescriptions, costing €226, at month 12. No costs relating to sick pay and inability to work were reported.

## Discussion

In the present study, we used data from two different sources to estimate the incidence and prevalence of HCC in Germany. Using the statutory health system database, which includes a sample of publically insured persons in Germany, the estimated annual crude incidence rate of HCC for 2013 was 12.17 per 100,000, whereas the annual crude incidence using relevant data from the Robert Koch Institute ranged from 7.18 to 8.71 per 100,000. The higher incidence rate in the statutory health system population as compared with the Robert Koch Institute estimates may be due in part to the predominantly urban population in the former sample, a trend noted in other reports of liver cancer incidence rates in rural and urban populations from Germany [22, 23]. In addition, we cannot be certain that we identified only incident cases of HCC in our analysis; however, we believe that a one-year period is sufficient to exclude patients with ongoing disease given the high mortality of this cancer. Differences in prevalence rates (23.89 per 100,000 in the GKV vs 12.25–14.85 per 100,000 in the Robert Koch Institute data [value directly calculated based on the Robert Koch Institute 10-year prevalence of liver cancer for 2013]) could have been because the statutory health system data are based on claims data and do not consider mortality, whereas the prevalence data from the Robert Koch Institute considered the patients who survived until 31 December 2013. Recalculating the Robert Koch Institute prevalence estimate considering all HCC cases over the course of 2013 resulted in a prevalence rate of 18.20–22.06 per 100,000 (Table 1), which is much closer to the GKV estimation. We further applied these data to estimate the number of patients in Germany with advanced HCC likely to be candidates for second-line therapy after prior sorafenib treatment. From a payer’s perspective in Germany, we estimated this number to range between 893 and 1390 patients per year based on the GKV and Robert Koch Institute data.

Epidemiological data from the present study also allowed us to characterise patients with advanced HCC likely to be candidates for second-line therapy after prior sorafenib. Of the 117 patients identified in our study with sufficient data for the follow-up analysis, most were men (85%), with a mean age of 72 years. Of the potential risk factors for HCC, liver cirrhosis (53.0%), often with accompanying fibrosis, and diabetes mellitus (56.4%) were observed most commonly; obesity and other metabolic disorders were also documented. The high prevalence of non-insulin-dependent diabetes in our population is consistent with systematic reviews and meta-analyses, which have reported that diabetes is associated with an increased risk of HCC [5, 24].

The German statutory health system included detailed real-life cost data, which allowed us to estimate costs for the target population from the perspective of the public healthcare provider. The mean total estimated healthcare costs accrued by the target population in the first year after sorafenib were €11,152 per patient. Mean monthly costs generally decreased over this time, which suggests that patients received more treatments and healthcare in the first few months following sorafenib therapy. For patients who were still alive at the end of the observation period (subgroup #4), mean total costs per patient for the first year after sorafenib discontinuation were €15,818, which were accounted for mainly by inpatient care (hospital stays) (€6272) and prescriptions (€8026). In patients who died during the observation period (subgroup #3) and likely were alive for a shorter period than patients in subgroup #4, total costs over the same timeframe were predictably lower (€9542), with inpatient care (€6556) accounting for most of the total. No costs relating to sick pay or inability to work were reported, possibly because most patients were retired and would have been receiving pension funds.

At the time of these analyses, no second-line therapies were approved for patients with HCC, representing an unmet need for patients who progress on sorafenib treatment. However, based on positive trial results, regorafenib (RESORCE trial [25]) was approved in both Europe and the USA in 2017, and nivolumab (CheckMate-040 trial [26]) was approved in the USA in 2017, for the second-line treatment of patients with HCC who had previously received sorafenib. In addition, a survival benefit versus placebo has been reported for ramucirumab following first-line treatment with sorafenib in patients with advanced HCC and elevated baseline alpha-fetoprotein levels (REACH-2 [27]) and for cabozantinib in patients with advanced HCC previously treated with sorafenib (CELESTIAL [28]). All studies evaluating potential second-line therapies had stringent inclusion criteria regarding adequate organ function, including liver function, and performance status. In the RESORCE trial, patients were also required to have documented radiographic progression and to have tolerated sorafenib [25]. These criteria, if applied in clinical practice, would likely limit the target population for second-line treatment.

### Limitations

The present study had several limitations, including the retrospective nature of the study design, the relatively small study sample and the limited medical information available from the database (e.g. information was not available on disease staging or treatment failure). For this reason, we needed to use an explorative approach to identify potential candidates for second-line therapy from the overall study population. In addition, data were not available concerning the status of patients with respect to their performance status and organ functioning. Because potential second-line therapies have only been evaluated in patients for whom these clinical factors were adequate, the numbers of patients eligible for post-sorafenib therapy may be lower than estimated. Our study sample was derived from the BKK health insurance scheme and included > 3 million persons insured with company health insurance in each index year. Although our source sample (the BKK database) was only ~ 5% of the total statutory healthcare population in Germany in 2013, basic demographic data suggested it was representative of the total insured population. Cost estimates may have had a slight downward bias because of incomplete coverage of services and incomplete cost data for ambulatory care. However, only about 5% of treatments charged in ambulatory care could not be linked with costs. A lack of more specific data meant we assumed sorafenib was used as first-line treatment, even though it appears cytotoxic chemotherapy was given instead in a few cases (2%).

## Conclusion

From a payer’s perspective in Germany, we estimate that approximately 893–1390 publicly insured patients with advanced HCC are potential candidates for second-line post-sorafenib therapy annually. The estimated total healthcare costs for this patient group are approximately €9500–16,000 per patient in the first year after completing sorafenib treatment. Hospital stays are the main contributing factor to these costs. These estimated costs will help decision makers determine the potential budgetary impact of new second-line therapies for advanced HCC.

## Additional files

Additional file 1: **Table S1**. Age and sex data on the pool of data from the German statutory health system and our study sample in 2013. (DOCX 19 kb)

Additional file 2: Supplementary information Exploratory analyses used to identify patients who failed sorafenib therapy. (DOCX 20 kb)

## Abbreviations

ACE: Angiotensin-converting enzyme
ATC: Anatomical therapeutic chemical
BCLC: Barcelona Clinic Liver Cancer
BKK: Betriebskrankenkasse
CI: Confidence interval
GKV: Gesetzliche Krankenversicherung
HCC: Hepatocellular carcinoma
ICD-10: *International Classification of Diseases and Related Health Problems, tenth revision*
SD: Standard deviation
