# Supplementary material for: Hepatocellular carcinoma after prior sorafenib treatment: incidence, healthcare utilisation and costs from German statutory health insurance claims data
Source: Health Econ Rev. 2018 Aug 27;8:18. doi: 10.1186/s13561-018-0199-1 (PMC6111015; doi:10.1186/s13561-018-0199-1)
Supplement: Supplementary file 1 — Table S1. Age and sex data on the pool of data from the German statutory health system and our study sample in 2013. (DOCX 19 kb) [file 13561_2018_199_MOESM1_ESM.docx]

**Additional file 1**

**Table S1** Age and sex data on the pool of data from the German statutory health system and our study sample in 2013.

|  | **Study sample** | | | | **German statutory health system** | | | |
| --- | --- | --- | --- | --- | --- | --- | --- | --- |
|  | **Men** | | **Women** | | **Men** | | **Women** | |
| **Age group (years)** | **N** | **%** | **N** | **%** | **N** | **%** | **N** | **%** |
| **0–14** | 218,880 | 6.5% | 208,051 | 6.2% | 4,531,267 | 6.5% | 4,299,636 | 6.2% |
| **15–19** | 93,110 | 2.8% | 83,867 | 2.5% | 1,819,247 | 2.6% | 1,727,541 | 2.5% |
| **20–24** | 113,267 | 3.4% | 98,083 | 2.9% | 2,054,709 | 2.9% | 2,013,699 | 2.9% |
| **25–29** | 115,878 | 3.5% | 105,045 | 3.1% | 2,189,472 | 3.1% | 2,202,582 | 3.2% |
| **30–34** | 114,711 | 3.4% | 110,858 | 3.3% | 2,130,992 | 3.1% | 2,206,508 | 3.2% |
| **35–39** | 107,574 | 3.2% | 102,338 | 3.1% | 1,913,143 | 2.7% | 2,053,248 | 2.9% |
| **40–44** | 124,333 | 3.7% | 116,613 | 3.5% | 2,196,783 | 3.1% | 2,403,461 | 3.4% |
| **45–49** | 155,603 | 4.7% | 143,041 | 4.3% | 2,791,638 | 4.0% | 3,046,201 | 4.4% |
| **50–54** | 149,744 | 4.5% | 134,263 | 4.0% | 2,741,793 | 3.9% | 2,994,359 | 4.3% |
| **55–59** | 120,631 | 3.6% | 107,277 | 3.2% | 2,292,658 | 3.3% | 2,550,527 | 3.7% |
| **60–64** | 101,250 | 3.0% | 94,490 | 2.8% | 2,011,830 | 2.9% | 2,330,784 | 3.3% |
| **65–69** | 78,092 | 2.3% | 70,447 | 2.1% | 1,542,753 | 2.2% | 1,840,717 | 2.6% |
| **70–74** | 89,096 | 2.7% | 80,896 | 2.4% | 1,858,108 | 2.7% | 2,306,326 | 3.3% |
| **75–79** | 71,470 | 2.1% | 70,096 | 2.1% | 1,535,213 | 2.2% | 2,069,764 | 3.0% |
| **80–84** | 39,033 | 1.2% | 46,890 | 1.4% | 840,565 | 1.2% | 1,345,667 | 1.9% |
| **85–89** | 19,799 | 0.6% | 33,222 | 1.0% | 413,523 | 0.6% | 942,924 | 1.3% |
| **90+** | 6,633 | 0.2% | 19,676 | 0.6% | 137,583 | 0.2% | 519,701 | 0.7% |
| **Total** | **1,719,104** | **51.4%** | **1,625,153** | **48.6%** | **33,001,277** | **47.2%** | **36,853,645** | **52.8%** |
|  | **3,344,257** | | | | **69,854,922** | | | |
