# Supplementary material for: Hepatocellular carcinoma after prior sorafenib treatment: incidence, healthcare utilisation and costs from German statutory health insurance claims data
Source: Health Econ Rev. 2018 Aug 27;8:18. doi: 10.1186/s13561-018-0199-1 (PMC6111015; doi:10.1186/s13561-018-0199-1)
Supplement: Supplementary file 2 — Supplementary information Exploratory analyses used to identify patients who failed sorafenib therapy. (DOCX 20 kb) [file 13561_2018_199_MOESM2_ESM.docx]

**Additional file 2**

**Supplementary information.** Exploratory analyses used to identify patients who failed sorafenib therapy

Radiologic examination (approximately 1.5–2 quarters after first sorafenib prescription) or symptomatic progression (approximately 1–1.5 quarters after) may be expected to identify failure of first-line therapy with sorafenib, given that reported median time to disease progression with sorafenib is approximately 5 months (Llovet et al. 2008). However, when seeking to identify patients with failed sorafenib therapy, it was not possible to use either symptomatic or radiologic progression because of missing or unspecific coding of radiologic examinations or symptoms in these secondary data. Furthermore sorafenib can be given despite serious adverse events because of the lack of therapy alternatives, and discontinuations of sorafenib therapy are therefore not always observable in a failure situation.

We therefore used an explorative approach that combined potential distinguishing markers to detect failure of first-line therapy. Characteristics or markers considered were the frequency and duration of sorafenib prescriptions, the pattern of sorafenib prescriptions, observation time and adverse events. Stratifications were set to examine potential influences of and differences between these specific characteristics or markers, all of which bear on the possibility of failure and were included to find an adequate definition of failure.

*Frequency and duration of prescriptions*

The mean number of prescriptions was 5.3 per patient (median of 3 prescriptions, due to rightward-skewed distribution). About 71% of the HCC patients with a sorafenib prescription (n=168) obtained >1 prescription of sorafenib, and 54% (n=127) obtained ≥3 prescriptions. The maximum number of prescriptions was 70. Differentiated by survival status, a lower number of prescriptions per patient was observed within the group of patients who already died.

The duration of sorafenib therapy was calculated as the difference between the date of the last sorafenib prescription and the first sorafenib prescription plus 28 days. The minimum duration of sorafenib therapy was 28 days by definition. The maximum observed therapy duration was about seven years.

Due to the rightward-skewed distribution of the duration of therapy, the median duration of sorafenib prescriptions was 96 days while the mean was 219 days. Duration of therapy was extended within the group of patients still alive compared to the group of patients who died during follow-up.

The median time between two prescriptions did not strongly differ with a rising number of prescriptions.

*Patterns of sorafenib prescriptions*

In a further approach the treatment pattern of sorafenib was differentiated by the type of application. A full dose was coded if there was only one prescription per patient or if there was ≤28 days between two prescriptions. The last dose was assumed to be equal to the previous dose (in situations where there was >1 prescription). A dose reduction was defined as a sorafenib prescription with >28 and ≤56 days until the following prescription. An interruption occurred if there was >56 days between prescriptions.

This could possibly be used as a marker for failure (because failure can depend on the number of reductions and interruptions). Cases of intolerance and cases of failure were considered with this approach. A disadvantage was that patients with only one prescription could not be included in this examination because at least two prescriptions were needed for a measurement of reduction or interruption. Therefore patients with only one prescription were excluded from reduction or interruption by definition (69 out of 237; 29%).

The number of full doses declined absolutely and relatively with the rising number of prescriptions per patient, especially within the first nine prescriptions. The reductions and, to a less pronounced extent, the interruptions also declined in their amount due to the shrinking group. Overall, the relative proportion of interruptions increased with the rising consecutive number of prescriptions.

There were 128 patients with ≥1 dose reduction, of whom, 95 died within the observation period. Of the 128 patients, 18% (n=23) had only one reduction, and 66% (n=85) up to three reductions. Furthermore 72 patients with at ≥1 interruption were identified, including 47 patients who died within the observation period. Of the 72 patients, 64% (n=46) had only one or two interruptions.

*Observation time*

Observation time was defined as the number of days from the first sorafenib prescription until death or end of follow-up at 31 December 2014. The average observation time was 320 days (range: 7 – 2548 days). Differentiating patients by the number of sorafenib prescriptions (1 vs. >1 prescription) led to a great divergence in observation time. Patients with >1 prescription had a longer observation time compared to those with only one. This gain was a median of >200 days (282 days vs. 55 days). Additionally, the observation time from the last prescription was examined due to relevance for a second-line therapy time frame after the end of sorafenib prescriptions. There was an observation time of median 70 days and mean 128 days. Stratified by the number of prescriptions there was a difference in mean and median. Patients with >1 prescription had a longer observation time (mean: 136 days, median: 78 days). It was accounted for that patients with one prescription had the same starting point in time for “from first” and “from last” prescription.

The descriptive statistics of observation time in days was additionally stratified by survival status. The difference between the observation time from the first prescription was enhanced if only those patients who died during the observation period were considered. Patients with >1 prescription had a median observation time of 231 days, whereas others achieved 53 days. The divergence was smaller between those who had >1 prescription in comparison to those who had only one with respect to the observation time from the last prescription (median 79 vs. 53 days).

A group of special interest consisted of those patients still alive at the end of the observational period and therefore with a long observation time after the last sorafenib prescription. A difference was observable in the median observation period from the first prescription in favour of patients with >1 prescription (+220 days). Concerning the last prescription, patients with >1 prescription also had a longer observation period than those with only one (157 vs. 130 days).

Further, we considered the observation time solely by classifying patients by the interval between the last sorafenib prescription and death or end of follow-up. The interval after the last sorafenib prescription was subdivided in accordance with the sorafenib treatment-guidelines into three sections: 1 – 28 days (full dose), ≥29 – 56 (dose reduction) and >56 days (interruption or failure).

Most patients “survived” >56 days after the last prescription. Death within 56 days occurred in 80 cases.

In addition to the summary statistics showing the distribution of observation time described above, the likelihood of survival starting from the first and the last prescription was analysed by the Kaplan-Meier method. The likelihood of surviving 102 days after the start of sorafenib therapy (first prescription) was 75% (median survival, approximately 233 days). The likelihood of survival from the last prescription for 39 days was 75% (median survival, 88 days). It was relevant that 69 patients had one prescription only; hence the survival time from last/first prescription was the same for these patients.

Analysis of observation time indicated a difference between sorafenib patients with only one prescription compared to those with >1 prescription. Examination of survival time via stratification in the Kaplan-Meier method also indicated statistically significant differences when time from first, but not last, prescription was considered. Both approaches, survival time from first and from last prescription, were stratified by number of prescriptions (1, ≥1) and compared with a log-rank test (first: p<0.0001; last p=0.2649).

*Occurrence of adverse events*

Adverse events with a severe characteristic can lead to a failure or a reduction or interruption of dose. Based on the sorafenib registration trial, the four most common high-grade adverse events are: i) hand-foot-skin reaction (L27.1 or L30.*), ii) diarrhoea (K52.9, K52.1 or A09.*), iii) hypertension (secondary, I15.*) and iv) abdominal pain (R10.*) (Llovet et al. 2008). Occurrence can be accounted for by at least one diagnosis or by a threshold of a minimum number of diagnoses per patient.

Adverse events were itemised by number of patients and number of diagnoses per patient. It should be noted that severity of the diagnoses was not recorded in the database. Stratification by number of prescriptions showed that with rising number of prescriptions the share of adverse events increased. Patients with only one prescription had the least share of adverse events, both overall and by particular events. Unsurprisingly, alive patients also had a larger share of adverse events than dead patients.

*Conclusions*

Different possibilities for an adequate failure definition were considered: number of prescriptions and duration of sorafenib therapy, pattern of prescriptions by definition of full dose, reduction and interruption of sorafenib therapy, observation time and adverse events. Upon the suspicion that number of prescriptions or survival status might be salient when identifying patients failing therapy, these two characteristics were partly used for stratification. The suspicion that patients with one prescription might be identified as a treatment failure was not verified and there was no larger share of adverse events in the stratum with one prescription.

Regarding the observation time in days classified by groups with a definition of full dose, reduction and interruption it was obvious that some patients were not eligible as “second-line” patients due to short observation time after the last sorafenib prescription (patients still alive at the end of the observation period). These patients defined by survival status are potential second-line patients.

*Reference*

Llovet JM, Ricci S, Mazzaferro V, et al.; SHARP Investigators Study Group. Sorafenib in advanced hepatocellular carcinoma. N Engl J Med. 2008;359:378-90.
